# Working Memory Performance for Differentially Conditioned Stimuli

**DOI:** 10.3389/fpsyg.2021.811233

**Published:** 2022-01-25

**Authors:** Richard T. Ward, Salahadin Lotfi, Daniel M. Stout, Sofia Mattson, Han-Joo Lee, Christine L. Larson

**Affiliations:** ^1^Center for the Study of Emotion and Attention, University of Florida, Gainesville, FL, United States; ^2^Department of Psychology, University of Florida, Gainesville, FL, United States; ^3^Department of Psychology, University of Wisconsin—Milwaukee, Milwaukee, WI, United States; ^4^Center of Excellence for Stress and Mental Health, VA San Diego Healthcare System, San Diego, CA, United States; ^5^Department of Psychiatry, University of California, San Diego, San Diego, CA, United States

**Keywords:** differential aversive conditioning, working memory capacity, working memory performance, threat-associated stimuli, safe-associated stimuli

## Abstract

Previous work suggests that threat-related stimuli are stored to a greater degree in working memory compared to neutral stimuli. However, most of this research has focused on stimuli with physically salient threat attributes (e.g., angry faces), failing to account for how a “neutral” stimulus that has acquired threat-related associations through differential aversive conditioning influences working memory. The current study examined how differentially conditioned safe (i.e., CS–) and threat (i.e., CS+) stimuli are stored in working memory relative to a novel, non-associated (i.e., N) stimuli. Participants (*n* = 69) completed a differential fear conditioning task followed by a change detection task consisting of three conditions (CS+, CS–, N) across two loads (small, large). Results revealed individuals successfully learned to distinguishing CS+ from CS– conditions during the differential aversive conditioning task. Our working memory outcomes indicated successful load manipulation effects, but no statistically significant differences in accuracy, response time (RT), or Pashler’s K measures of working memory capacity between CS+, CS–, or N conditions. However, we observed significantly reduced RT difference scores for the CS+ compared to CS– condition, indicating greater RT differences between the CS+ and N condition vs. the CS– and N condition. These findings suggest that differentially conditioned stimuli have little impact on behavioral outcomes of working memory compared to novel stimuli that had not been associated with previous safe of aversive outcomes, at least in healthy populations.

## Introduction

Humans are bombarded with visual stimuli throughout their daily routines, with most of this information being irrelevant to current task goals. Due to the limited capacity of cognitive resources (Desimone and Duncan, 1995; Corbetta and Shulman, 2002), we must selectively attend to stimuli most relevant to our ongoing tasks (Yantis, 2000; Theeuwes, 2010) and those that provide critical information pertaining to contingencies within the environment (Miskovic and Keil, 2012). This enhanced attentional processing of important information impacts downstream cognitive systems (Hillyard et al., 1998), such as working memory (Awh et al., 2006; Ikkai and Curtis, 2011; Gazzaley and Nobre, 2012). Working memory is a limited-capacity system that supports the representation and manipulation of information over a short interval (Cowan, 2001, 2010, 2017; Baddeley, 2012), and is critical for carrying out ongoing tasks. Because the capacity of working memory is finite, an interaction between top-down and bottom-up processes occurs in which individuals selectively attend to goal-relevant stimuli while inhibiting task irrelevant information (Yantis, 2000; Theeuwes, 2010). However, salient features present in affective stimuli, whether task-relevant or irrelevant, can drive bottom-up attentional processes and in turn influence cognitive systems (McMains and Kastner, 2011; Sussman et al., 2016), such as working memory.

While an abundance of work has examined the impact of affective stimuli on working memory processes in anxious populations (see Moran, 2016; Gambarota and Sessa, 2019; Schweizer et al., 2019), others have also found such effects in healthy individuals (see Lindström and Bohlin, 2011; Pe et al., 2013; Xie et al., 2017; Smith et al., 2018; Schweizer et al., 2019; Sugi et al., 2020). Specifically, a set of recent meta-analyses by Schweizer et al. (2019) found that the influence of affective vs. neutral stimuli on working memory depends largely on the valence of the stimuli, and whether such stimuli are task relevant or irrelevant. Their results indicated that in healthy populations task-relevant affective stimuli enhanced working memory accuracy, regardless of stimulus valence. In contrast, negatively valenced target stimuli yielded increased response times (RTs) compared to positively valenced target stimuli. These outcomes provide a broad sense of the influence that affective stimuli have on working memory processes in healthy populations. However, categories of negatively valenced stimuli differ in their salience (e.g., threatening vs. disgusting), likely yielding variability in the impact such stimuli have on cognitive systems (Brosch et al., 2010), such as working memory.

Given that attentional biases, specifically toward threat-related stimuli, serve as a potential risk factor for the development and maintenance of anxiety (Bar-Haim et al., 2007; Ouimet et al., 2009; Heeren et al., 2013; Van Bockstaele et al., 2014), it is essential to clarify the impact that threat-related stimuli have on working memory systems. Prior work has shown that threat-related stimuli are preferentially stored in working memory over neutral stimuli, regardless of whether they are task-relevant (Reinecke et al., 2006, 2010; Jackson et al., 2008, 2009, 2014; Sessa et al., 2011; Stout et al., 2013, 2020; Meconi et al., 2014) or irrelevant (Stout et al., 2013; Ye et al., 2018). In addition, others have found that threat-related stimuli negatively impact RTs (Kensinger and Corkin, 2003; Sessa et al., 2011; Stout et al., 2013) while increasing accuracy (Simione et al., 2014) compared to neutral stimuli. Taken together, these findings suggest that threat-related stimuli are stored to a greater degree and improve accuracy relative to neutral stimuli, but at the expense of increased RTs. However, thus far, this body of research has focused exclusively on biologically inherent threat-related stimuli (e.g., angry faces, spiders, etc.), failing to account for how a simple stimulus that has gained threat-related attributes influences working memory.

Threat-related attributes are often acquired through experience, where a specific neutral cue becomes associated with an aversive event, thus eliciting a fearful response when presented with this now conditioned cue. For example, a doorbell ring may become associated with a loud bark from a dog resulting in a startled response. After several pairings of this doorbell-bark contingency, one may feel startled when hearing a doorbell ring even if a dog is not present to bark. This example demonstrates the elements of aversive conditioning, in which a neutral cue is repeatedly presented with an aversive event, leading to an association between this cue and the aversive event. This association results in the cue becoming an aversive conditioned stimulus, or a CS+, such that it now elicits a fearful or defensive response. Major theoretical models of anxiety emphasize the role of aversive learning in the etiology of anxiety disorders and fear responses (Lissek et al., 2005; Delgado et al., 2006; Boddez et al., 2013; Duits et al., 2015; Fullana et al., 2016, 2020). Specifically, much of this work has found that anxious individuals demonstrated enhanced conditioning acquisition (Lissek et al., 2005) and impaired extinction (Barrett and Armony, 2009; Duits et al., 2015), or the ability to learn that a CS+ no longer is associated with an aversive outcome after several presentations of the CS+ without an aversive outcome. While this body of work is important, less remains known regarding how these threat-conditioned stimuli influence higher order cognitive systems, especially given that previous reports have found evidence indicating that these systems alter aversive conditioning processes (Raes et al., 2009; Fani et al., 2012; Stout et al., 2018).

Like other threat-related stimuli, stimuli that have acquired threat-related attributes through conditioning (i.e., CS+) are likely to alter basic cognitive processes. Evidence supporting this view can be found through behavioral (Koster et al., 2004; Haddad et al., 2011; Notebaert et al., 2011; Schmidt et al., 2015a; Dowd et al., 2016) and eye tracking studies (Mulckhuyse et al., 2013; Schmidt et al., 2015b; Hopkins et al., 2016; Nissens et al., 2017), in which a CS+ was found to bias attentional processes, such that attention is preferentially deployed toward these stimuli. While these biases in attention were observed for CS+ relative to neutral stimuli (Notebaert et al., 2011; Schmidt et al., 2015b; Hopkins et al., 2016), others also demonstrated such effects between CS+ and a stimulus that was associated with safety, or a CS–, finding greater attentional biases toward CS+ compared to CS– (Koster et al., 2004; Mulckhuyse et al., 2013; Schmidt et al., 2015a; Dowd et al., 2016; Hopkins et al., 2016; Nissens et al., 2017). These results suggest that CS+ are salient and likely enhance bottom-up processes, and are more likely to bias attentional processing compared to CS–. The results of these attentional alterations are prone to influence downstream cognitive processes, such as working memory. However, to our knowledge no study thus far has investigated the impact CS+ stimuli have on working memory storage compared to CS– and neutral stimuli.

The current study aimed to address this gap in the literature by examining how simple stimuli that have acquired threat (i.e., CS+) or safe (i.e., CS–) attributes influence working memory processes using a differential aversive conditioning task followed by a change detection task. Specifically, participants were first presented with two types of rectangles differing in color, one associated with an aversive shock (i.e., CS+) and another that had no shock association (i.e., CS–). Next, they completed a change detection task in which these now CS+ and CS– stimuli were presented in addition to a novel stimulus that had not been associated with either threat or safe attributes (i.e., N). This design allowed for the development of learned contingencies for simple stimuli, and the evaluation of how these now CS+ and CS– stimuli impact working memory performance and storage.

Based on previous literature indicating enhanced storage of threat-related stimuli in working memory (Reinecke et al., 2006, 2010; Jackson et al., 2008, 2009, 2014; Sessa et al., 2011; Stout et al., 2013, 2020; Meconi et al., 2014), we predicted greater behavioral estimates of working memory capacity, measured through Pashler’s K scores (Rouder et al., 2011), for the CS+ conditions compared to the CS– and N conditions. Most prior attentional work with conditioned stimuli has focused on CS+ compared to CS– or CS+ vs. neutral stimulus contrasts. Although Kim and Anderson (2021) found that saccade response times toward a neutral target stimulus with a CS– distracter were faster than toward a CS– target with a neutral distracter, it is difficult to tease apart attentional deployment toward a CS– compared to neutral target stimulus given the addition of the distracter stimuli present in their design. Because little to no work, to our knowledge, has examined attentional or behavioral differences between CS– and neutral target stimuli, we did not have any directional *a priori* hypotheses concerning working memory capacity differences between these conditions.

Given these hypotheses concerning working memory storage, we also hypothesized a similar pattern of results concerning task accuracy, with greater accuracy for CS+ vs. CS– and N conditions. This idea is also supported from previous work indicating affective target stimuli yield increased task performance (Schweizer et al., 2019), and follows the logic that higher accuracy will be associated with greater storage of target stimuli (i.e., Pashler’s K scores). Finally, we predicted longer RTs for CS+ compared to CS– and N conditions based on previous reports indicating attentional biases for such stimuli (Koster et al., 2004; Mulckhuyse et al., 2013; Schmidt et al., 2015a; Dowd et al., 2016; Hopkins et al., 2016; Nissens et al., 2017). As described prior, a paucity of work, if any, has examined behavioral performance between CS– and neutral or novel, non-associated target stimuli in attentional or working memory tasks, leading us to make no *a priori* predictions for these condition comparisons. No effects or interactions between condition or load were expected.

In addition to our primary hypotheses, we also computed behavioral difference scores for accuracy, RT, and Pashler’s K scores. Specifically, this examined the change in these behavioral measures between the N and conditioned stimuli (i.e., CS+ and CS–) conditions. Similar to our main predictions, we anticipated greater working memory storage for CS+ compared to CS– stimuli relative to the N condition, reflected by a greater positive Pashler’s K difference score for CS+ compared to CS–. Furthermore, we anticipated that accuracy would show a similar pattern, with greater CS+ accuracy compared to the CS– stimuli relative to the N condition, quantified by a greater positive accuracy difference score for CS+ compared to CS–. Finally, we expected greater increases in in RT for the CS+ relative to N condition vs. the CS– relative to N condition, indicated by an increased CS+ RT difference score compared to the CS– RT difference score.

## Materials and Methods

The study design and analyses were preregistered^1^ prior to data collection. The preregistration originally planned to include electroencephalography (EEG) measures, but these data were unable to be collected due to experimental restrictions resulting from the COVID-19 pandemic. As such, all analyses are limited to behavioral data. In addition, a novel, non-associated stimulus condition was added to the final experimental design to serve as a “neutral” comparison condition between CS+ and CS– conditions.

### Power Analyses

Power analyses were conducted using G*Power (Faul et al., 2007, 2009) assuming a small effect size (η*_*p*_*^2^ = 0.02), a power of 0.8, and an α level of 0.05 prior to data collection. Initial power analyses reported in our preregistration pertained to differences between CS+ and CS– conditions, with a within-subject design resulting in the use of a 2 (Load; small, large) × 2 (Condition; CS+, CS–) repeated measures ANOVA as our inferential statistical analyses. This power analysis indicated a required sample size of 69 participants.

We conducted a secondary power analysis with the inclusion of a novel, non-associated stimulus condition (i.e., N), resulting in a 2 (Load; small, large) × 3 (Condition; CS+, CS–, N) repeated measures ANOVA with the same parameters described above. The outcome of this analysis suggested a sample size of 54 participants. Because we were primarily interested in specific differences between CS+ and CS– conditions relative to their differences with the N condition (i.e., a 2 × 2 design), we aimed to recruit a total of 69 participants.

### Participants

Eighty-six undergraduates were recruited from the University of Wisconsin—Milwaukee to participate in the study in exchange for course credit or $10 cash. Participants were at least 18 years old and had no history of visual or neurological impairments. Seventeen participants were excluded from data analysis due to study withdrawal (*n* = 1), poor task accuracy (i.e., < 70%; *n* = 2), technical errors during task administration (*n* = 1), and failure to learn the CS contingency (*n* = 13). This resulted in a total of 69 (47 Female; *M*_*age*_ = 21.91, *SE* = 0.64) participants (2 American Indian or Alaskan Native, 2.9%; 6 Asian or Pacific Islander, 8.7%; 4 African American, 5.8%; 11 Hispanic, 15.9%; 43 White, 62.3%, 1 Other, 1.4%, and 2 No Answer Provided, 2.9%) were used for data analyses.

### Materials and Procedure

#### Shock Administration

Participants were attached to electrical stimulation (i.e., shock) hardware using Psychlab’s SHK1 Pain Stimulation Shocker (Contact Precision Instruments, Cambridge, MA) following completion of informed consent. Shocks were delivered through two electrodes placed approximately 2 inches above participants’ right ankle using double-sided tape and conductive gel. An initial shock work-up was conducted to determine individualized shock levels that participants considered as “painful, but tolerable.” During the shock work-up, participants were informed that they would receive a mild electrical shock, and were asked to rate it on a scale from 1 to 9 (e.g., 1 meaning “you can’t feel it at all” and 9 meaning that “it is painful, but tolerable”). Once participants’ individualized level 9 was reached, that parameter was set for the experiment. To prevent habituation to the shocks over the course of the study, the experimenter asked the participant if they still rated the shock at the ideal level of 9 after completion of the differential aversive conditioning task and following each between-block conditioning in the change detection task. If the participants rated the shock below a level 9, then the parameter would be adjusted until that rating was met before proceeding with the experiment. All but 18 (∼26%) participants increased their shock at least once throughout the duration of the experiment.

#### Differential Aversive Conditioning Task

**FIGURE 1:**
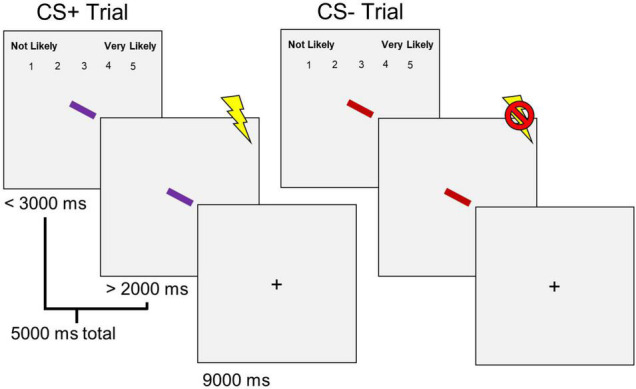
Differential aversive conditioning task. Sixteen total trials (8 CS+, 8 CS–) were presented in a pseudorandomized order. CS+ trials co-terminated with a shock, and CS– trials did not.

Participants completed a differential aversive conditioning task (Figure 1) following the shock work-up. The task consisted of 16 trials: 8 for the CS+ and 8 for the CS– conditions. All trials were pseudorandomized to prevent two consecutive trials of the same condition from occurring. Each trial began with a colored rectangle presented at a central fixation. In addition, a 1–5 rating scale at the top of the screen asked participants to indicate the likelihood of receiving a shock when viewing the presented stimulus (1 being “Not Likely” and 5 being “Very Likely”) was presented simultaneously with the rectangle. The rating scale was presented for 2,000 ms or until participants made a response before disappearing. The rectangle remained on the screen before co-terminating with a shock (CS+), delivered during the final 500 ms, or no shock (CS–). Thus, the rectangle was presented for a total duration of 5,000 ms. Trials were separated by an ITI of 9,000 ms. Each rectangle was assigned a specific color (i.e., purple, red, or green—luminance matched at ∼68 cd/m^2^) based on the respective condition (i.e., CS+, CS–, or N). The N colored rectangle was only presented after the differential aversive conditioning task. The rectangle was presented in any of four orientations (vertical, horizontal, left 45°, and right 45°), and condition colors were counterbalanced across participants.

After completing the task, participants were randomly presented with each of the colored rectangles (one at a time) in a random orientation along with 2 prompts: One asking them to rate their anxiety toward the presented rectangle on a scale from 1 to 9 (1 being “Not Anxious” and 9 being “Very Anxious”) and the other requiring them to indicate the likelihood of receiving a shock when viewing the presented rectangle on a scale from 1 to 5 (1 being “Not Likely” and 5 being “Very Likely”). Each prompt remained on the screen until a response was made.

Participants’ learning of the CS contingency was assessed by taking the average online shock likelihood ratings for the final two CS+ and CS– trials for each condition, respectively, and comparing these two values. If the average of the final two trials’ online shock likelihood rating in the CS– condition’s was greater than or equal to the final two trials’ online shock likelihood rating in the CS+ condition, the participant was removed from data analysis due to failure to explicitly learn the CS contingencies (i.e., *n* = 13).

#### Change Detection Task

**FIGURE 2:**
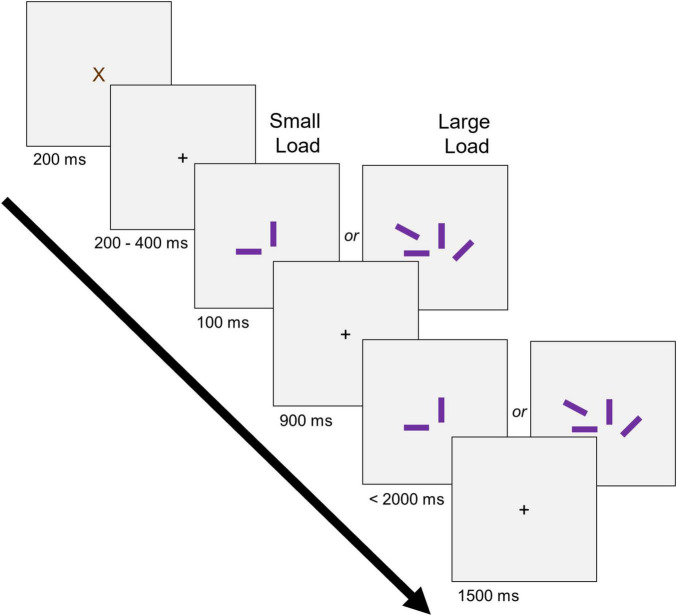
Change detection task. Two hundred and forty trials were presented in a randomized order containing 3 conditions (CS+, CS–, N) across 2 loads (small, large).

Following conditioning, participants completed a change detection task (Figure 2) to assess working memory performance and storage capacity (Eng et al., 2005; Rouder et al., 2011; Xu et al., 2018; Feuerstahler et al., 2019) between the CS+, CS–, and N conditions. Each trial began with a brown “X” (200 ms) followed by a central fixation cross (200–400 ms). Next, randomly oriented rectangles (vertical, horizontal, left 45°, and right 45°) were briefly (100 ms) presented in a central stimulus array (total array size = 5.07° × 5.46°; left/right of fixation = 2.53°; above/below fixation = 2.73°) before an empty delay period (900 ms). Following this delay, the rectangles were re-presented (< 2,000 ms), and participants were required to indicate on a keyboard with their right hand whether or not there was a 45°change in orientation within one of the rectangles (“1” for no change, and “2” for a change). Trials were separated by a 1,500 ms inter-trial interval.

Prior to the test portion of the task, participants completed a practice session consisting of 12 trials: Six in a small Load (i.e., 2 rectangles) and six in a large Load (i.e., 4 rectangles). All rectangles in the practice session were colored black. Following the practice session, participants completed the test portion of the change detection task in which the previously CS+, CS–, and N colors were used. The task consisted of 3 primary Conditions (i.e., CS+, CS–, N) across two Loads (i.e., small, 2 rectangles; large, 4 rectangles) for a total of 240 trials, with 40 trials per Condition and Load size (e.g., 40 for CS+ 2, 40 for CS+ 4, etc.). Trials were separated across 4 blocks, with each block containing 60 trials.

Between blocks, participants were presented with each Condition by Load combination, and asked to rate their anxiety and shock likelihood to the stimuli using the scales described above. Next, a brief between-block conditioning session (i.e., 4 total trials; 2 CS+ and 2 CS– randomly presented) occurred following the differential aversive conditioning trial procedure, only excluding the shock likelihood prompt. These between-block procedures were conducted to prevent potential extinction of the learned CS associations, and validate that the CS+, CS–, and N stimuli still elicited their desired shock likelihood and anxiety levels. After this brief conditioning session, participants were given the same anxiety and shock likelihood prompts seen at the end of the differential aversive conditioning task described above. Overall, participants took approximately 55 min to complete the differential aversive conditioning task, the practice change detection task, and the full change detection task.

RT data was inspected at the individual trial-level. Similar to our previous work (Ward et al., 2019, 2020, 2021), we removed trials with RTs below 150 ms and incorrect trials. This was done to remove any trials that may reflect random responding, trials where no response was made, and to directly assess RTs for correct-only trials.

### Statistical Analyses

Significant interactions and main effects were decomposed using Bonferroni corrected pairwise comparisons. All analyses used Greenhouse-Geisser adjustments when Sphericity assumptions were violated, and BF_10_ were reported for all non-significant results in our analyses to assess the strength of evidence for the null hypothesis.

#### Differential Aversive Conditioning

The dependent variables for the differential aversive conditioning task included online shock likelihood, post-task shock likelihood, and post-task anxiety ratings. A 2 (CS+, CS–) × 8 (Trial) repeated measures ANOVA was conducted for online shock likelihood ratings, and two one-way repeated measures ANOVAs with three-levels (CS+, CS–, N) were used to assess post-task shock likelihood and anxiety ratings.

#### Change Detection Task

Primary dependent variables for the change detection task included accuracy (% correct), response time (RT) in ms, and Pashler’s K score. Separate 2 (Load) × 3 (Condition) ANOVAs were conducted for these variables. Pashler’s K formula [i.e., K = N × (HR–FA)/(1–FA)] was used as a behavioral estimate of working memory capacity (Rouder et al., 2011) based on the proportion of correct responses made if a target changed orientation (i.e., HR or hit rate), and proportion of incorrect responses made if a target did not change orientation (i.e., FA or false alarms).

Our secondary dependent variables were difference scores for accuracy, RT, and Pashler’s K scores for each Load. Each variable’s difference scores were calculated using the following formulas: Accuracy difference score = (N–CS) × –1; RT difference score = N–CS; Pashler’s K difference score = CS–N. Using these formulas, greater difference score values reflect greater accuracy, faster RTs, and greater working memory storage capacity for the given CS condition (i.e., CS+, CS–) relative to the N condition in that specific load (i.e., small or large). Separate 2 (CS+, CS–) × 2 (Load) ANOVAs were used to examine difference scores between CS+ and CS– conditions.

We also examined between block conditioning shock likelihood and anxiety ratings using two separate one-way ANOVAs with three-levels (CS+, CS–, N). This was done to ensure that the between-block conditioning was successful in maintaining the learned CS contingencies throughout the change detection task.

### Bayes Factor Analyses

Bayes Factor analyses, specifically using Bayes Factor 10 (BF_10_) values, were conducted for all non-significant outcomes to evaluate the degree of evidence for the null vs. alternative hypothesis (Dienes, 2014, 2016; Jarosz and Wiley, 2014; Lee and Wagenmakers, 2014; Wagenmakers et al., 2016, 2018a,b; Keysers et al., 2020; Lakens et al., 2020; van Doorn et al., 2021). Although BF_10_ values are treated on a continuous scale, with values closer to 0 reflecting stronger evidence for the null hypothesis (Lee and Wagenmakers, 2014; Wagenmakers et al., 2016, 2018a,b; Keysers et al., 2020; van Doorn et al., 2021), guidelines for communicating discrete BF_10_ outcomes have been proposed (see Jeffreys, 1939; Kass and Raftery, 1995; Jarosz and Wiley, 2014), with van Doorn et al. (2021) suggesting the following interpretations for BF_10_ values: 0–0.1, strong evidence for null hypothesis; 0.1–0.33, moderate evidence for null hypothesis; 0.33–1, weak evidence for null hypothesis; 1–3, weak evidence for alternative hypothesis; 3–10, moderate evidence for alternative hypothesis; and > 10, strong evidence for alternative hypothesis. We used this guideline as a formality for communicating our confidence in arriving at a null outcome, as was done in our previous work (Ward et al., 2019, 2020, 2021). For outcomes with strong support for the alternative hypothesis held, we further examined these effects through follow-up comparisons to determine whether such effects were present.

## Results

### Differential Aversive Conditioning Task

#### Online Shock Likelihood

**FIGURE 3:**
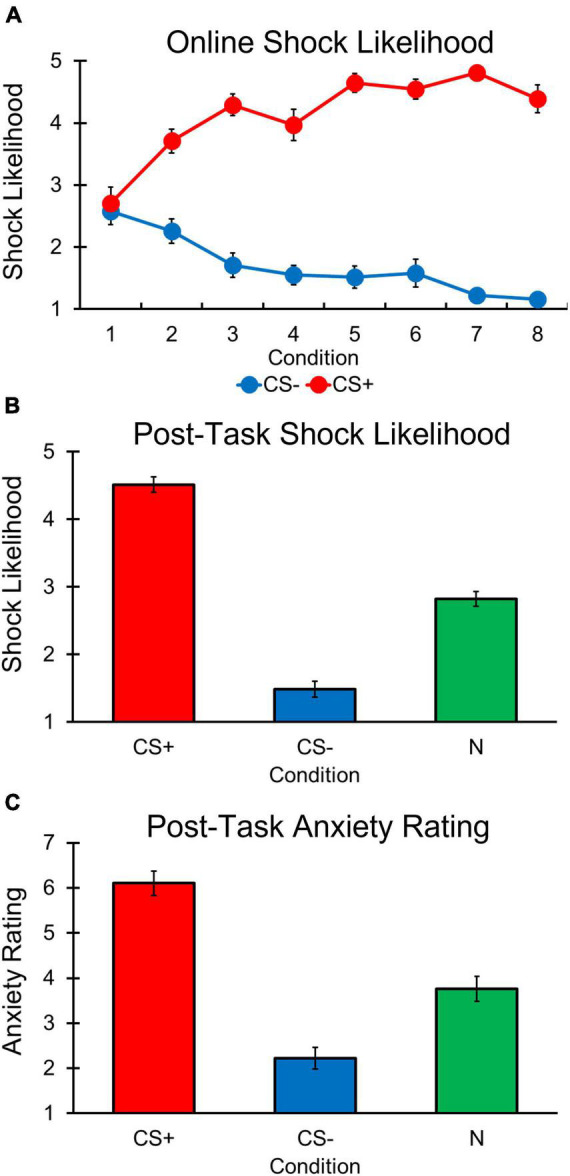
Differential aversive conditioning results. Error bars represent standard errors. **(A)** Online shock likelihood ratings for CS+ stimulus were greater (i.e., more likely to predict a shock) than the CS– stimulus following trial 1 in each condition. **(B)** Post-task shock likelihood ratings were higher for CS+ stimulus compared to N and CS– stimuli. Ratings were also higher for the N than CS– stimuli. **(C)** Post-task anxiety ratings were greater for CS+ compared to N and CS– stimuli. Ratings were also lower for the CS– than N stimuli.

A main effect of Condition [*F*(1, 30) = 191.352, *p* < 0.001, η*_*p*_*^2^ = 0.864], was observed with participants reporting greater shock likelihood for the CS+ compared to CS– condition (Figure 3A). In addition, a significant main effect of Trial was observed, [*F*(7, 210) = 2.191, *p* = 0.036, η*_*p*_*^2^ = 0.068]. However, *post hoc* comparisons showed that online shock likelihood ratings did not significantly differ between any of the trials (all *p*’s > 0.392).

Our main effects of Trial and Condition were explained by a significant Condition by Trial interaction [*F*(4, 322, 129, 671) = 20.216, *p* < 0.001, η*_*p*_*^2^ = 0.403], with follow-up comparisons revealing that online shock likelihood ratings between CS+ and CS– conditions did not differ on trial 1 [*t*(49) = –0.333, *p* = 0.741], but significantly differed on the remaining 7 trials [trial 2, *t*(48) = –3.824, *p* < 0.001; trial 3, *t*(63) = –11.350, *p* < 0.001; trial 4, *t*(63) = –10.410, *p* < 0.001; trial 5, *t*(63) = –14.100, *p* < 0.001; trial 6, *t*(66) = –13.100, *p* < 0.001; trial 7, *t*(65) = –27.820, *p* < 0.001; trial 8, *t*(66) = –18.150]. Thus, participants learned the CS contingencies following the first trial of each condition.

#### Post-task Shock Likelihood and Anxiety

Post-conditioning shock likelihood ratings revealed a main effect of Condition [*F*(1, 626, 110, 537) = 151.003, *p* < 0.001, η*_*p*_*^2^ = 0.690; Figure 3B], with participants reporting greater shock likelihood for CS+ vs. CS– [*t*(68) = 14.254, *p* < 0.001] and N [*t*(68) = 11.019, *p* < 0.001] conditions. Participants also reported greater shock likelihood for the N condition compared to the CS– condition, [*t*(68) = –8.853, *p* < 0.001]. A similar pattern was observed for post-task anxiety ratings [*F*(2, 136) = 60.867, *p* < 0.001, η*_*p*_*^2^ = 0.472; Figure 3C] in which participants reported greater anxiety for the CS+ compared to the CS– [*t*(68) = 9.890, *p* < 0.001] and N [*t*(68) = 7.591, *p* < 0.001] conditions, and greater anxiety for the N condition compared to the CS– condition was also found, [*t*(68) = –4.322, *p* < 0.001]. These results suggest that following differential aversive conditioning, participants were aware the CS+ was associated with a shock and it induced greater anxiety compared to the CS–, and that the N was neither predictive of shock nor safe likelihood and reflected a level of anxiety between the safe and threat-associated stimuli.

### Change Detection Task

#### Accuracy

**FIGURE 4:**
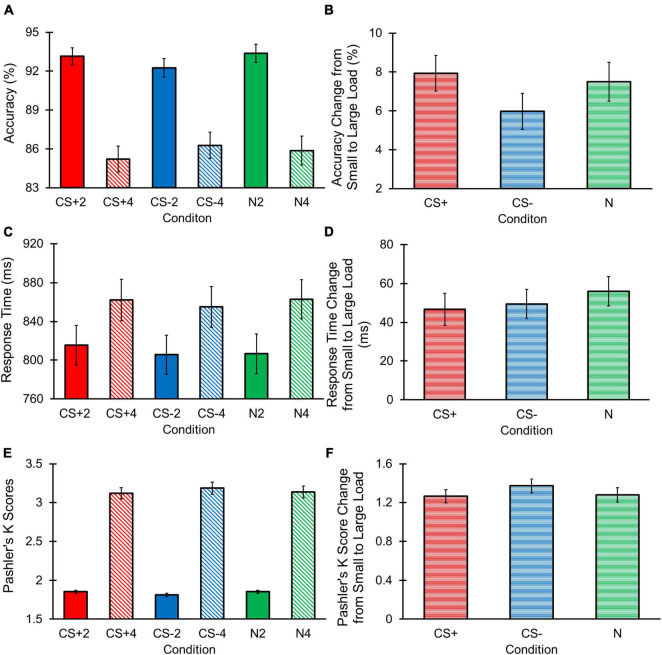
Change detection task raw behavioral results. Error bars represent standard errors. **(A)** Accuracy was greater for small vs. large loads. No main effect of Condition or Condition by Interaction was found. **(B)** No significant effects of Condition were observed for changes in accuracy across loads. **(C)** RTs were longer for large vs. small loads. No main effect of Condition or Condition by Load interaction was found. **(D)** No significant effects of Condition were observed for changes in RT across loads. **(E)** Pashler’s K scores were larger for large vs. small loads. No main effect of Condition or Condition by Load interaction was found. **(F)** No significant effects of Condition were observed for changes in Pashler’s K score across loads.

The repeated measures ANOVA for accuracy (Figure 4A) revealed a main effect of Load [*F*(1, 68) = 101.992, *p* < 0.001, η*_*p*_*^2^ = 0.600] with greater accuracy for the small vs. large load. However, we did not observe a main effect of Condition [*F*(2, 136) = 0.440, *p* = 0.645, η*_*p*_*^2^ = 0.006, BF_10_ = 0.031], with BF_10_ outcomes providing strong evidence for the null hypothesis. In addition, the Condition by Load interaction was non-significant, [*F*(2, 136) = 1.746, *p* = 0.179, η*_*p*_*^2^ = 0.025, BF_10_ = 2.837 × 10^38^]. Despite this non-significant result, the BF_10_ value indicated strong evidence for the alternative hypothesis, prompting us to further deconvolve this interaction.

In further investigating this interaction, we did not observe a main effect of Condition across the small [*F*(2, 136) = 1.972, *p* = 0.143, η*_*p*_*^2^ = 0.028, BF_10_ = 0.277] or large [*F*(2, 136) = 0.763, *p* = 0.468, η*_*p*_*^2^ = 0.011, BF_10_ = 0.100] loads, with the BF_10_ outcome indicating strong support for the null hypotheses. Final comparisons examined each condition’s difference in accuracy between the small and large loads (i.e., change in accuracy from a small to large load for each condition). Results found that the difference in accuracy between a small and large load did not differ based on Condition, [*F*(2, 136) = 1.746, *p* = 0.178, η*_*p*_*^2^ = 0.025, BF_10_ = 0.236; Figure 4B]. Given that the BF_10_ results for these follow-up analyses suggested weak to strong support for the null hypotheses in our analyses, we conclude that there is unlikely to be a Condition by Load interaction for accuracy.

#### Response Time

The repeated measures ANOVA for RT (Figure 4C) revealed a main effect of Load [*F*(1, 68) = 70.555, *p* < 0.001, η*_*p*_*^2^ = 0.509] with longer RTs in the large compared to small load. However, we did not observe a main effect of Condition [*F*(2, 136) = 1.742, *p* = 0.79, η*_*p*_*^2^ = 0.025, BF_10_ = 0.065], and the BF_10_ outcome suggested strong evidence for the null hypothesis. Although we failed to observe a significant Condition by Load interaction [*F*(2, 136) = 0.635, *p* = 0.526, η*_*p*_*^2^ = 0.009, BF_10_ = 6.166 × 10^22^], the BF_10_ results provided strong evidence for the alternative hypothesis. Thus, we conducted follow-up comparisons to investigate this potential interaction effect.

No significant main effect of Condition across the small [*F*(2, 136) = 1.726, *p* = 0.182, η*_*p*_*^2^ = 0.025, BF_10_ = 0.224] or large [*F*(2, 136) = 0.849, *p* = 0.430, η*_*p*_*^2^ = 0.012, BF_10_ = 0.104] loads was found, and respective BF_10_ values indicated strong support for these null hypotheses. Next, we examined each condition’s difference in RT between the small and large loads, in which we found non-significant differences in RT change between conditions, [*F*(2, 136) = 0.635, *p* = 0.532, η*_*p*_*^2^ = 0.009, BF_10_ = 0.087; Figure 4D]. Given the weak to strong evidence for our null hypotheses revealed through the BF_10_ values, we interpreted these outcomes as supporting the unlikelihood that there was a Condition by Load interaction for RT.

#### Pashler’s K Score

The repeated measures ANOVA for Pashler’s K score, a behavioral measure of working memory storage, revealed a main effect for Load [*F*(1, 68) = 70.555, *p* < 0.001, η*_*p*_*^2^ = 0.509] with greater K scores in the large compared to small load (Figure 4E). A non-significant main effect of Condition [*F*(2, 136) = 0.063, *p* = 0.939, η*_*p*_*^2^ = 0.001, BF_10_ = 0.027] was observed, and the BF_10_ results indicated strong evidence for the null hypothesis. We also did not observe a significant Condition by Load [*F*(2, 136) = 1.553, *p* = 0.216, η*_*p*_*^2^ = 0.022, BF_10_ = 6.731 × 10^115^] interaction, but the BF_10_ outcomes indicated strong evidence for the alternative hypothesis. As such, we conducted follow-up comparisons to deconvolve this interaction.

Follow-up examinations revealed a non-significant main effect for Condition across the small [*F*(2, 136) = 2.675, *p* = 0.073, η*_*p*_*^2^ = 0.038, BF_10_ = 0.511], and large [*F*(2, 136) = 0.592, *p* = 0.555, η*_*p*_*^2^ = 0.009, BF_10_ = 0.084] loads. Our BF_10_ results suggested strong evidence for the null hypothesis examining the large load, and weak evidence for the null hypothesis concerning the small load. However, because there was no evidence for the alternative hypothesis for the small load, we did not further examine this effect. We examined each condition’s difference in K scores between the small and large loads. Difference in K scores across loads also produced a non-significant main effect of Condition [*F*(2, 136) = 1.553, *p* = 0.215, η*_*p*_*^2^ = 0.022, BF_10_ = 0.192; Figure 4F], with BF_10_ results providing strong support for the null hypothesis. Thus, there is unlikely to be a Condition by Load interaction effect for Pashler’s K scores.

#### Accuracy Difference Score

**FIGURE 5:**
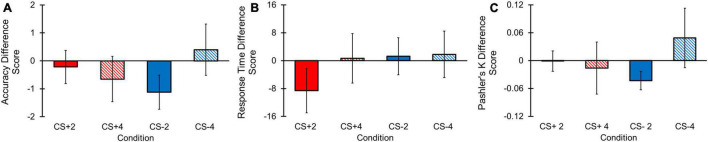
Change detection task behavioral difference score results. Error bars represent standard errors. **(A)** No main effects for Condition or Load, or a Condition by Load interaction effect were observed for accuracy difference scores. **(B)** No main effects for Load or a Condition by Load interaction effect were observed for RT difference scores. A main effect of Condition was found, with greater RT difference scores for CS+ vs. CS– conditions. **(C)** No main effects for Condition or Load, or a Condition by Load interaction effect were observed for Pashler’s K difference scores.

Accuracy difference scores, reflecting the relative difference in accuracy between each CS condition compared to the N condition for each load, did not yield a significant main effect for Condition [*F*(1, 68) = 0.020, *p* = 0.888, η*_*p*_*^2^ < 0.001, BF_10_ = 0.138], Load [*F*(1, 68) = 0.295, *p* = 0.589, η*_*p*_*^2^ = 0.004, BF_10_ = 0.353], or a Condition by Load interaction [*F*(1, 68) = 3.540, *p* = 0.064, η*_*p*_*^2^ = 0.049, BF_10_ = 0.011; Figure 5A]. Although BF_10_ values for the Condition and Condition by Load effects revealed strong and moderate strengths for the null hypotheses, respectively, our outcomes for the Condition effect indicated weak evidence for the null hypothesis. However, because there was also no evidence for the alternative hypothesis, we opted not to further investigate the Load main effect. Overall, these results suggest that accuracy difference scores between CS+ and CS– conditions were not significantly different from one another, regardless of load.

#### Response Time Difference Score

Response Time (RT) difference scores were examined to determine if the CS+ condition demonstrated greater difference in RT relative to the N condition compared to the CS– condition relative to the N condition. Results revealed a main effect for Condition [*F*(1, 68) = 4.084, *p* = 0.047, η*_*p*_*^2^ = 0.057] with a lower RT difference score for the CS+ compared to CS– condition (Figure 5B). We did not find a significant main effect for Load [*F*(1, 68) = 1.091, *p* = 0.300, η*_*p*_*^2^ = 0.029, BF_10_ = 0.349] or a Condition by Load interaction [*F*(1, 68) = 0.114, *p* = 0.737, η*_*p*_*^2^ = 0.001, BF_10_ = 0.034]. BF_10_ values revealed strong evidence for the null hypothesis in the Condition by Load analysis, but weak evidence for the null hypothesis for the main effect of Load. However, follow-up comparisons for the Load main effect were not conducted given that the BF_10_ value suggested no evidence for the alternative hypothesis. These results indicate the CS+ condition yielded significantly lower RT difference score compared to the CS– difference score, implying that the CS+ condition resulted in longer RTs in the CS+ compared to N condition than the RTs between the CS– and N conditions.

#### Pashler’s K Difference Score

Pashler’s K difference scores, or the difference score between a CS+ /CS– and N condition Pashler’s K score in each load, respectively, was examined to determine whether CS+ and CS– conditions differed in their degree of working memory storage relative to the N condition. No significant main effects for Condition [*F*(1, 68) = 0.102, *p* = 0.751, η*_*p*_*^2^ = 0.001, BF_10_ = 0.143], Load [*F*(1, 68) = 0.499, *p* = 0.482, η*_*p*_*^2^ = 0.007, BF_10_ = 0.222], or a Condition by Load [*F*(1, 68) = 2.426, *p* = 0.124, η*_*p*_*^2^ = 0.034, BF_10_ = 0.010] interaction for Pashler’s K difference scores were found (Figure 5C). Furthermore, our BF_10_ outcomes provided strong evidence for the null hypotheses in these analyses. Therefore, differences in behavioral estimates of working memory capacity between CS+ and CS– conditions relative to the N condition were non-significant.

#### Post-between Block Conditioning Shock Likelihood and Anxiety

**FIGURE 6:**
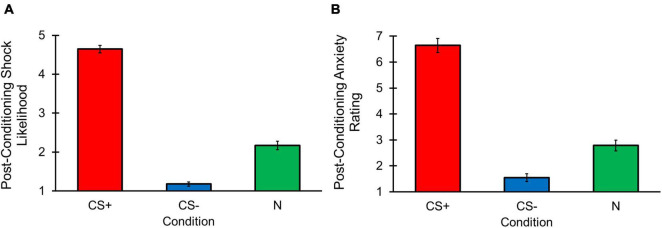
Change detection task post-between block conditioning shock likelihood and anxiety results. Error bars represent standard errors. **(A)** Post-between block conditioning shock likelihood ratings were greater for the CS+ stimulus compared to N and CS– stimuli. Ratings were also higher for the N than CS– stimulus. **(B)** Post-between block conditioning anxiety ratings were higher for the CS+ compared to N and CS– stimuli. The N stimulus also had greater anxiety ratings compared to the CS– stimulus.

Post-between block conditioning shock likelihood revealed a main effect of Condition [*F*(1, 613, 109, 705) = 344.835, *p* < 0.001, η*_*p*_*^2^ = 0.835; Figure 6A], with greater shock likelihood ratings for the CS+ compared to the CS– [*t*(68) = 26.515, *p* < 0.001] and N [*t*(68) = 15.090, *p* < 0.001] conditions. The CS– condition also had lower shock likelihood ratings than the N condition, [*t*(68) = –9.251, *p* < 0.001]. Post-between block conditioning anxiety ratings also demonstrated a main effect of Condition [*F*(1, 626, 110, 537) = 151.003, *p* < 0.001, η*_*p*_*^2^ = 0.690; Figure 6B], with higher levels of anxiety reported for the CS+ vs. CS– [*t*(68) = 16.262, *p* < 0.001] and N [*t*(68) = 12.296, *p* < 0.001] conditions, and lower anxiety for the CS– condition compared to N condition, [*t*(68) = –6.792, *p* < 0.001]. These outcomes suggest that the learned CS associations remained intact and were maintained throughout the change detection task.

## Discussion

Affective stimuli influence working memory processes, even in healthy individuals (Lindström and Bohlin, 2011; Pe et al., 2013; Xie et al., 2017; Smith et al., 2018; Schweizer et al., 2019; Sugi et al., 2020), with such effects often being exacerbated for threat-related stimuli (Kensinger and Corkin, 2003; Reinecke et al., 2006, 2010; Jackson et al., 2008, 2009, 2014; Sessa et al., 2011; Stout et al., 2013, 2020; Meconi et al., 2014; Simione et al., 2014). However, this body of work has focused exclusively on inherently, biologically threatening stimuli, failing to account for how simple stimuli that have acquired threat-related attributes through learning contingencies influence working memory storage. In addition, the impact of learned safety cues on working memory is unknown. Therefore, we aimed to investigate how simple stimuli that had been conditioned to achieve either safe (i.e., CS–) or threat-related (i.e., CS+) attributes influenced behavioral markers of working memory storage.

We found that participants successfully learned threat vs. safe stimulus-associated contingencies throughout the differential aversive conditioning task. Specifically, individuals rated the CS+ as predictive of an electrical shock, and the CS– as predictive of no electrical shock. In addition, on average participants learned these contingencies after the first trial presentation of the CS+ and CS–. Further post-task shock likelihood responses demonstrated that the CS+ was rated as being most likely to predict a shock, followed by the novel, non-associated stimulus (i.e., N), and with the CS– not being predictive of a shock. These outcomes suggest that participants were not only able to differentiate CS+ and CS– based on their shock contingencies, but that the newly presented N stimulus held a degree of uncertainty, being neither fully predictive of a shock or lack of shock. Our post-task anxiety ratings matched this pattern of results, with the CS+ eliciting greater anxiety than the N and CS–. Similar to our post-task shock likelihood ratings, the N stimulus was rated as eliciting an anxiety level between the CS– and CS+. Therefore, participants successfully learned that the CS+ was associated with a shock, the CS– was associated with no shock, and were uncertain about the N stimulus, viewing this stimulus as in-between the CS+ and CS–.

For working memory outcomes, contrary to our hypotheses, we found no evidence for differences in our behavioral estimates of working memory capacity, measured through Pashler’s K scores, between the CS+, CS–, or N conditions. This same pattern of results was observed for accuracy, failing to support our hypothesis concerning this dependent measure. Furthermore, the difference scores for Pashler’s K scores and accuracy between each CS condition and the N condition did not differ between CS+ and CS– conditions. Our Bayes Factor analyses also yielded weak to strong evidence for the null hypotheses in our analyses, supporting the notion that these conditions did not differ in terms of accuracy or Pashler’s K difference scores. While it’s possible that these stimuli lost their threat and safe attributes over the course of the change detection task, the between block shock likelihood and anxiety assessments suggest that participants retained these contingencies throughout the task. Taken together, these results suggest that behavioral markers of performance (i.e., accuracy) and working memory storage (i.e., Pashler’s K scores) are not influenced by simple stimuli that have threat and safe-related attributes learned through previous conditioning. Thus, with respect to our primary question, conditioned threat stimuli were not stored to a greater degree in working memory compared to conditioned safe stimuli.

These findings contradict prior reports that demonstrated enhanced working memory capacity for threat-related stimuli (Reinecke et al., 2006, 2010; Jackson et al., 2008, 2009, 2014; Sessa et al., 2011; Stout et al., 2013, 2020; Meconi et al., 2014). However, it is important to consider the differences between the current study and these previous reports. First, these studies utilized stimuli containing threatening facial expressions (Jackson et al., 2008, 2009, 2014; Sessa et al., 2011; Stout et al., 2013, 2020; Meconi et al., 2014) or spiders (Reinecke et al., 2006, 2010). Such stimuli are likely to have greater salience in that they hold biologically inherent threat relevance and may differentially influence attention and working memory functions compared to simple stimuli that have recently acquired threat-related associations. For instance, Öhman et al. (2012) suggested that some forms of threat-related stimuli are more likely to induce attentional capture than other forms of threatening stimuli based on their evolutionary relevance. In line with this idea, Soares et al. (2014) found that snakes were more rapidly detected compared to spiders, potentially due to evolutionary mechanisms increasing our ability to detect more threatening and predatory stimuli. Facial stimuli also hold evolutionary value for social interactions, such as submissive or competitive behaviors (Öhman et al., 2012), and others have found that facial stimuli are prioritized regardless of task-relevant or attentional demands (Lavie et al., 2003; Reddy et al., 2004), leading to their rapid and efficient processing (Holmes et al., 2005, 2009; Mogg et al., 2008; Öhman et al., 2012) due to dedicated neural networks for such stimuli (Pitcher et al., 2009; Pourtois et al., 2013). As such, it is possible that facial stimuli, and other biologically threatening stimuli, are processed differently than simple stimuli that have recently gained threat-related attributes. Thus, although participants learned the threat and safety stimulus associations, and felt greater levels of anxiety toward the CS+ stimulus relative to the CS– and N stimuli, these conditioned stimuli recently gained these threat and safety attributes and may not hold the same degree of evolutionary value as other threatening stimuli, such as faces or spiders.

Second, it may be the case that behavioral measures of working memory storage are not sensitive to detect whether CS+ and CS– stimuli are differentially stored in working memory compared to N stimuli in a change detection task. For example, previous work also failed to identify behavioral differences in working memory storage, despite observing neural markers indicating alterations in the storage and maintenance of stimuli in working memory (Basten et al., 2012; Meconi et al., 2014; Qi et al., 2014a,b; Ward et al., 2020). In line with these findings, others have also criticized the use of behavioral markers when assessing other cognitive processes, such as RT, compared to the use of more temporally precise neural measures (Kappenman et al., 2014, 2015, 2021). Importantly, Kappenman et al. (2021) argued that distinct attentional processes are confounded in behavioral measures, likely contributing to their poor reliability and lack of consistency across studies. Similarly, it may be the case that significant differences in the storage of conditioned CS+ and CS– stimuli are unlikely to be observed using accuracy or Pashler’s K scores, and instead are only observable under the lens of psychophysiological and neuroimaging techniques.

Finally, although our change detection task design was similar to others, our task included more trials per condition than these previous studies (Jackson et al., 2008, 2014; Reinecke et al., 2010), potentially yielding greater signal-to-noise estimates of behavioral accuracy and working memory storage. However, others (Reinecke et al., 2006; Jackson et al., 2009; Sessa et al., 2011; Stout et al., 2013, 2020; Meconi et al., 2014) employed a similar, or even larger (i.e., for event-related potential studies), trial count and found effects for threat-related stimuli. Therefore, we do not believe this factor contributed to the differences between our study and previous reports.

Our RT outcomes also did not support our hypotheses for longer RTs for the CS+ and CS– conditions compared to the N condition, and longer RTs for the CS+ than CS– condition. Instead, RTs did not significantly differ between CS+, CS–, and N conditions across each load, with our Bayes Factor analyses providing evidence for these null effects. These outcomes conflict with previous behavioral reports of longer RTs for CS+ compared to CS– stimuli (Koster et al., 2004; Dowd et al., 2016; Kappenman et al., 2021), and when a CS+ was presented as a distracter compared to a CS– (Schmidt et al., 2015a). Despite the non-significant findings from our primary RT analyses, we did observe significant RT difference scores between CS+ and CS– conditions across both loads. Specifically, participants showed greater slowdowns in RT from the N condition to the conditioned stimuli for CS+ compared to CS– conditions. Thus, although we failed to observe significant differences between each stimulus condition, we did find that CS+ and CS– conditions differed in the degree of their change in RT relative to the N condition. These outcomes suggest that attentional processing of these stimuli was impacted to some degree, although we are unable to determine whether this was isolated to the encoding or probe phases, and whether attentional selection or suppression processes were influenced. Therefore, future work should incorporate temporally precise neuroimaging measures to decipher which stages of attentional processing are likely to be influenced by the presence of CS+ stimuli on working memory tasks.

Nonetheless, it is important to note several points concerning our results, given the novelty of using conditioned CS+ and CS– target stimuli. First, we found significant load effects for our primary analyses concerning accuracy, RT, and Pashler’s K scores. Specifically, small loads (i.e., 2 rectangles) resulted in greater accuracy, faster RTs, and lower Pashler’s K scores compared to large loads (i.e., 4 rectangles). These findings suggest that the addition of these conditioned stimuli did not prevent the typical expected load effects one would observe in a canonical change detection task from occurring (e.g., Luck and Vogel, 1997; Vogel et al., 2001; Eng et al., 2005; Zhou and Thomas, 2015), and also replicate previous work that used valenced stimuli in these task designs (Sessa et al., 2011; Stout et al., 2013, 2015; Ward et al., 2019, 2021). Furthermore, because all condition colors were matched for luminance, our task consisted of simple shapes as used in classical change detection task paradigms (Luck and Vogel, 1997; Vogel et al., 2001; Feuerstahler et al., 2019), and our stimuli were only manipulated based on their valence attribute from previous conditioning (i.e., CS+ and CS–) or novel presentation (i.e., N), we believe our task design to be sound given that no other task parameters were significantly altered.

It is possible that the effects of conditioned stimuli on working memory processes are only present in clinically anxious or individuals with elevated sub-clinical anxiety, and are absent in healthy populations. For example, Schweizer et al.’s (2019) series of meta-analyses indicated negligible effect size estimates of affective stimuli on behavioral outcomes in working memory tasks. In contrast, effect sizes were larger in individuals with mental health problems. Thus, it is possible that stimuli that acquire threat and safe attributes through experience are more likely to impact working memory in individuals experiencing clinical and sub-clinical levels of psychopathology. However, future work is needed to examine the impact conditioned stimuli have on these processes in clinical and elevated sub-clinical populations before strong conclusions regarding this issue can be made.

The number trials required for transfer effects to a cognitive task should also be considered. For instance, previous studies examining the impact of reward-associations on working memory have employed significantly greater acquisition trials prior to assessing working memory performance (Gong and Li, 2014; Infanti et al., 2015). However, other behavioral studies examining the impact of conditioned stimuli on cognitive tasks assessing attentional processes (Haddad et al., 2011; Notebaert et al., 2011; Schmidt et al., 2015a; Dowd et al., 2016) used a similar range of trials per conditioned stimulus during their acquisition phase while still observing significant effects on attention. Alternatively, it is possible that extinction effects occurred for our CS+ stimuli after the initial trials of each block. Given that each block consisted of 60 trials prior to between-block differential conditioning reinforcement trials, it may be the case that participants became extinguished to the CS+ after the first presentations of this condition, regardless of set size. Despite this possibility, others (Notebaert et al., 2011; Dowd et al., 2016) have presented over 90 trials per block, with Schmidt et al. (2015a) including 180 total trials (36 per block) in a visual search task without any reinforcement of the CS+ while still observing effects on attentional processes. Furthermore, the randomization of our trial conditions within each block could have resulted in some subjects only being presented as few as ∼6 CS+ presentations across the initial 20 trials of a block, unlikely leading to these extinction effects. Given this design and the findings of previous behavioral work (Haddad et al., 2011; Notebaert et al., 2011; Schmidt et al., 2015a; Dowd et al., 2016), we find it unlikely that participants experienced extinction effects, at least not to the extent where it would have differentially impacted behavioral performance.

Finally, it is important to mind the influence cognitive load has on affective processing in relation to differential aversive conditioning processes (Raes et al., 2009; Stout et al., 2018; Laing et al., 2019; de Voogd and Phelps, 2020). Specifically, previous reports have indicated working memory load moderates the association between anxiety and differential aversive conditioning acquisition (Laing et al., 2019). Others have also found that extinction learning is enhanced in those with greater working memory abilities for larger loads (Stout et al., 2018; although see de Voogd and Phelps, 2020). Although some have reported reduced extinction effects resulting from increased working memory loads (Raes et al., 2009), these results highlight a pattern of findings reflecting a complex interaction between cognitive load and affective processing, specifically related to differential aversive conditioning processes. As such, it is possible that the increased loads used in our working memory task attenuated the effects of the conditioned stimuli. Further research examining the potential interaction between these factors is warranted to further unravel the influence cognitive load has on the processing of conditioned threat and safety cues, specifically in a working memory task.

Overall, our results suggest that differentially conditioned stimuli do not influence the degree of working memory storage, accuracy, or processing speed (i.e., RT) compared to novel, non-associated stimuli. These non-significant differences in behavioral outcomes are supported by Bayes Factor analyses, suggesting there are unlikely to be differences in our dependent variables between these stimulus conditions. However, we did find that changes in RT between conditioned and neutral stimuli were greater for the CS+ condition than CS– condition. Specifically, the CS+ condition showed slower RTs compared to the N condition, and this change in RT was significantly greater than the difference in RT between the CS– and N conditions. This suggests that some alterations in attentional processing likely occurred between CS+ and CS– stimuli in relation to the novel, non-associated stimulus. However, we are unable to deconvolve the specific effects on attention given the use of behavioral data alone. The non-significant differences observed in the current study may be due to the type of threat-related stimuli used, such that more biologically inherent threatening stimuli are more likely to influence working memory storage and performance. In addition, it may also be the case that healthy participants do not experience alterations in working memory processes when conditioned stimuli are used. Thus, the current study suggests that differentially conditioned simple stimuli included yield minimal effects on behavioral working memory outcomes when serving as targets in a change detection task.

## Data Availability Statement

The raw data supporting the conclusions of this article will be made available by the authors, without undue reservation.

## Ethics Statement

The studies involving human participants were reviewed and approved by the University of Wisconsin—Milwaukee Institutional Review Board. The patients/participants provided their written informed consent to participate in this study.

## Author Contributions

RW contributed to the study design, data collection, analyses, manuscript drafting, and supervised the project. SL and DS contributed to the study design, manuscript development, and analyses. SM contributed to the study design, manuscript development, and supervised the project. H-JL and CL contributed to the study design and manuscript development. All authors contributed to the article and approved the submitted version.

## Conflict of Interest

The authors declare that the research was conducted in the absence of any commercial or financial relationships that could be construed as a potential conflict of interest.

## Publisher’s Note

All claims expressed in this article are solely those of the authors and do not necessarily represent those of their affiliated organizations, or those of the publisher, the editors and the reviewers. Any product that may be evaluated in this article, or claim that may be made by its manufacturer, is not guaranteed or endorsed by the publisher.

## Footnotes

1
https://osf.io/j9846/
